# Minor salivary gland mucoepidermoid carcinoma in children and adolescents: a case series and review of the literature

**DOI:** 10.1186/1752-1947-6-182

**Published:** 2012-07-03

**Authors:** Priyanshi Ritwik, Kitrina G Cordell, Robert B Brannon

**Affiliations:** 1 Department of Pediatric Dentistry, Louisiana State University Health Sciences Center School of Dentistry, 1100 Florida Avenue, Box 139, New Orleans, LA 70119, USA; 2 Division of Oral and Maxillofacial Pathology, Louisiana State University Health Sciences Center School of Dentistry, 1100 Florida Avenue, New Orleans, LA 70119, USA

## Abstract

**Introduction:**

Because well-documented cases of mucoepidermoid carcinomas that are of minor salivary gland origin and occur in children and adolescents have rarely been reported, little information regarding their clinical features and biologic behavior is available. This case report represents a retrospective clinical analysis of five minor salivary gland mucoepidermoid carcinomas accessioned from a 35-year period at the Louisiana State University School of Dentistry and combines the data with 15 well-documented cases from the English language literature.

**Case presentation:**

The five mucoepidermoid carcinomas in patients from birth to 19 years of age accounted for 1.3% of the accessioned minor salivary gland neoplasms. There were an additional 15 well-documented cases in the literature. Combining the data for the 20 mucoepidermoid carcinomas resulted in a mean age of 13.5 years and a 2.3:1 female-to-male ratio. Collectively, the hard palate, soft palate, and hard palate/soft palate junction accounted for 85% of the cases. Thirty-five percent of the cases presented as a fluctuant submucosal swelling with surface color alterations. The average duration was five months, and bone involvement occurred in seven cases. A histologic grade of low to intermediate predominated (95%). Surgical removal was the treatment in all cases. Thirteen cases had adequate follow-up of three years or more, and recurrence was documented in only one case. There were no cases of death or metastasis in this series.

**Conclusions:**

In children and adolescents, mucoepidermoid carcinomas have a female predilection and occur most commonly on the hard or soft palate or both. A fluctuant submucosal lump with a bluish color is a helpful diagnostic clue. The histologic grades of most mucoepidermoid carcinomas in the first and second decades of life are low and, to a lesser degree, intermediate. Complete surgical excision is the treatment of choice and results in a recurrence rate of less than 10%.

## Introduction

The relative frequency of epithelial salivary gland tumors occurring in children and adolescents ranges from 3.7% to 5.5% [1]. Moreover, the high incidence of malignancy in minor salivary gland tumors is well established. Batsakis [2], in a review of the literature, determined that 52.3% of minor salivary gland tumors were malignant. Similar findings, of from 44% to 65%, have been reported [3-8]. Therefore, the overall incidence of minor salivary gland neoplasms is low in the pediatric-adolescent age group, but in view of their high incidence of malignancy, their importance should not be underestimated.

The most common type of malignant salivary gland neoplasm of epithelial (parenchymal) origin in the pediatric-adolescent age group is the mucoepidermoid carcinoma (MEC) [9]. Other types of minor salivary gland adenocarcinomas in this age group are rarely reported. Most of the malignant neoplasms are found in the parotid gland; only a few pediatric and adolescent cases have been well documented in the minor glands [9]. In fact, a review of the English language literature revealed only 15 well-documented cases of MEC of minor salivary gland origin in this age group [1,10-19]. A search of the Louisiana State University School of Dentistry (LSUSD) Division of Oral and Maxillofacial Pathology archives of a 35-year period supports the paucity of malignant intraoral salivary gland tumors and the reported frequency of MECs in the first two decades of life. Therefore, the purposes of this retrospective analysis were to investigate the clinical features and biologic behavior of minor salivary gland MECs occurring in children and adolescents (from birth to 19 years of age) from the LSUSD and to compare these findings with well-documented cases reported in the English language literature.

In this study, the age of the pediatric-adolescent population was from birth to 19 years, which represents the first two decades of life. Minor salivary gland MECs from this age group were selected from the LSUSD Department of Oral and Maxillofacial Pathology archives of the period of 1 January 1969 to 31 December 2004. The Louisiana State University Health Sciences Center (LSUHSC) Institutional Review Board (#6450) approved the research protocol. Demographic and clinical information was recorded for each case, and the histopathologic slides were reviewed by one of the authors (RBB). The LSUSD MECs were histologically graded by using the criteria set forth by Auclair *et al*. [20]. The LSUHSC School of Public Health Louisiana Tumor Registry provided follow-up information for the LSUSD cases.

A search of the English language medical and dental literature was performed for well-documented minor salivary gland MEC cases in the pediatric and adolescent age group. The search was carried out in Medline for ‘mucoepidermoid carcinoma and salivary gland carcinoma’, and limits were set to human subjects under 19 years and the English language. The search was last updated in September 2011. Once studies were identified, individual articles and their references were checked for additional studies. It should be noted that although investigators have published numerous series on salivary gland neoplasms, they provide an age range only. They do not correlate the age of the patient to the location of the lesion or offer other demographic and clinical information, such as the identity of the specific minor salivary gland involved. Some of these series did indicate that at least one patient was in the first or second decade of life; however, we did not include them in this study, because they lacked adequate detail [8,21-37]. Central (intraosseous) MECs of the maxilla and mandible were not included in this study.

## Case presentation

### Prevalence

A total of 396 minor salivary gland neoplasms were accessioned at LSUSD from the 35-year period. Of these 396 cases, 14 (3.5%) were benign and malignant neoplasms from patients in the first or second decade of life. Five (35.7%) of the 14 neoplasms were malignant; all were MECs.

A search of the English language literature revealed 15 well-documented cases of MEC in the minor salivary glands of children younger than 19 years of age [1,10-19]. These data were combined with the information from the five LSUSD cases. Table 1 displays a summary of the demographics, clinical findings, treatment, and follow-up of the 15 well-documented minor salivary gland MECs in the literature, in addition to the five new LSUSD cases.

**Table 1:** Mucoepidermoid carcinomas

**Source**	**Age, years**	**Gender**	**Site**	**Duration**	**Size, cm**	**Bone/LN Involvement**	**Histologic grade**	**Treatment**	**Recurrence/Interval**	**Follow-up**
Tipton [10]	1.5	Male	Buccal mucosa	NS	1.0	NS	Poorly differentiated	Wide local excision	No	NED 6 years
Budnick [11]	12	Female	Hard palate	NS	2.0	Bone: NSLN: NS	Low	Excision down to bone	No	NED 3 years
Gustafsson *et al*. [12]	13	Female	Hard palate	2 months	1.5	Bone: NoLN: NS	Low	Resection of the tumor	No	NED 1.5 years
Lack and Upton [13]	18	Female	Upper lip^a^	NS	NS	Bone: NSLN: NS	Low to focally intermediate	Near total maxillectomy	No	NED 8 years
Fonseca *et al*. [1]	14	Male	Soft palate	NS	NS	Bone: NSLN: No	Low	1st surgery2nd surgery	Yes/5 years	NED, NS
Fonseca *et al*. [1]	14	Female	Soft palate	NS	NS	Bone: NSLN: No	Low	Surgery	No	NED 6 months
April *et al*. [14]	10	Female	Junction of the hard/soft palate	Nasal congestion × 1 month	0.5	Bone: erosion into left naresLN: No	Intermediate	NS	NS	NS
Aguiar *et al*. [15]	13	Female	Junction of the hard/soft palate	7 months	3.0	Bone: NoLN: No	NS	Excision	No	NED 4 months
Winslow *et al*. [16]	10	Female	Buccal mucosa	NS	NS	Bone: NoLN: No	Intermediate	Wide local excision	NS	NS
Flaitz [17]	8	Male	Hard palate	9 months	2.0	Bone: NoLN: No	Low	Wide local excision down to periosteum	NS	NS
Caccamese and Ord [18]	17	Male	Left junction of the hard/soft palate	NS	1.5	Bone: NoLN: No	Low	Local resection	No	NED 94 months
Caccamese and Ord [18]	14	Female	Hard palate	1 month	1.0	Bone: NoLN: No	Low to intermediate	Excision	No	NED 62 months
Caccamese and Ord [18]	22^b^	Male	Palate, NOS	7 years	2.0	Bone: NoLN: No	Low	Excision with 1.0cm margins	No	NED 34 months
Caccamese and Ord [18]	16	Female	Junction of the soft palate and anterior pillar	1 year	2.5	Bone: NoLN: No	Low to intermediate	Local resection	No	NED 42 months
Moraes *et al*. [19]	14	Female	Left hard palate	1 year	5	Bone: YesLN: No	Low	Transoral resection	No	NED 4 years
LSUSD	15	Female	Left hard palate	3 weeks	NS	Bone: NoLN: No	Low	Excision	No	NED 21 years
LSUSD	14	Female	Right hard palate	NS	3.0	Bone: YesLN: NS	Low	Excision	No	NED 19 years
LSUSD	15	Female	Left hard palate	NS	NS	Bone: NSLN: NS	Low	NS	No	NED 9 years
LSUSD	18	Female	Left hard palate	3 weeks	NS	Bone: NSLN: NS	Low	Excision	No	NED 8 years
LSUSD	19	Male	Right junction of the hard/soft palate	‘Forever’	2.0	Bone: NoLN: NoLungs: No	Low to intermediate	Wide surgical excision with ‘alveolar bone biopsy’	No	NED 2 years

^a^Reported as ‘buccal sulcus anteriorly in the premaxillary area’. ^b^Lesion followed for seven years (since the age of 15). LSUSD, Louisiana State University School of Dentistry; LN, lymph node; NED, no evidence of disease; NOS, not further specified; NS, not stated.

### Summary of findings

**Figure 1:**
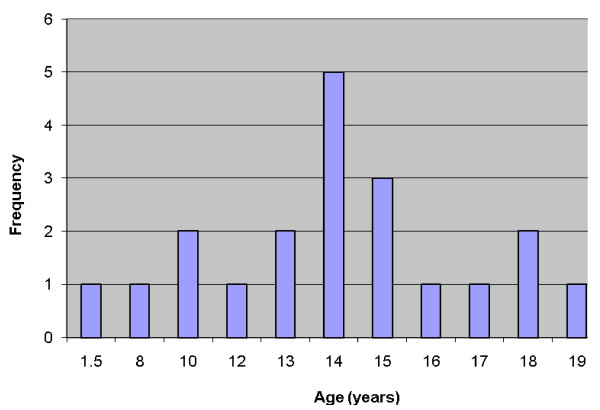
Age distribution of patients with mucoepidermoid carcinoma.

**Figure 2:**
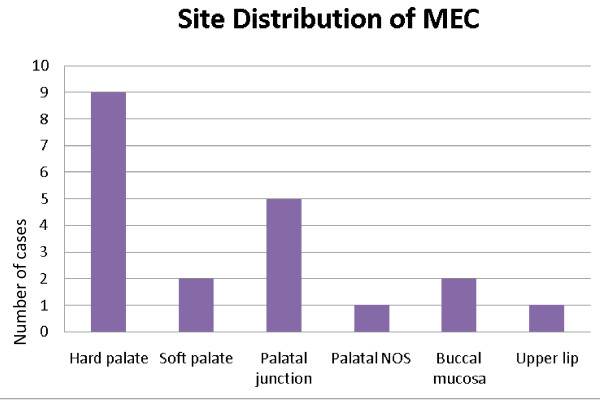
Site distribution of mucoepidermoid carcinoma in children and adolescents.

**Figure 3:**
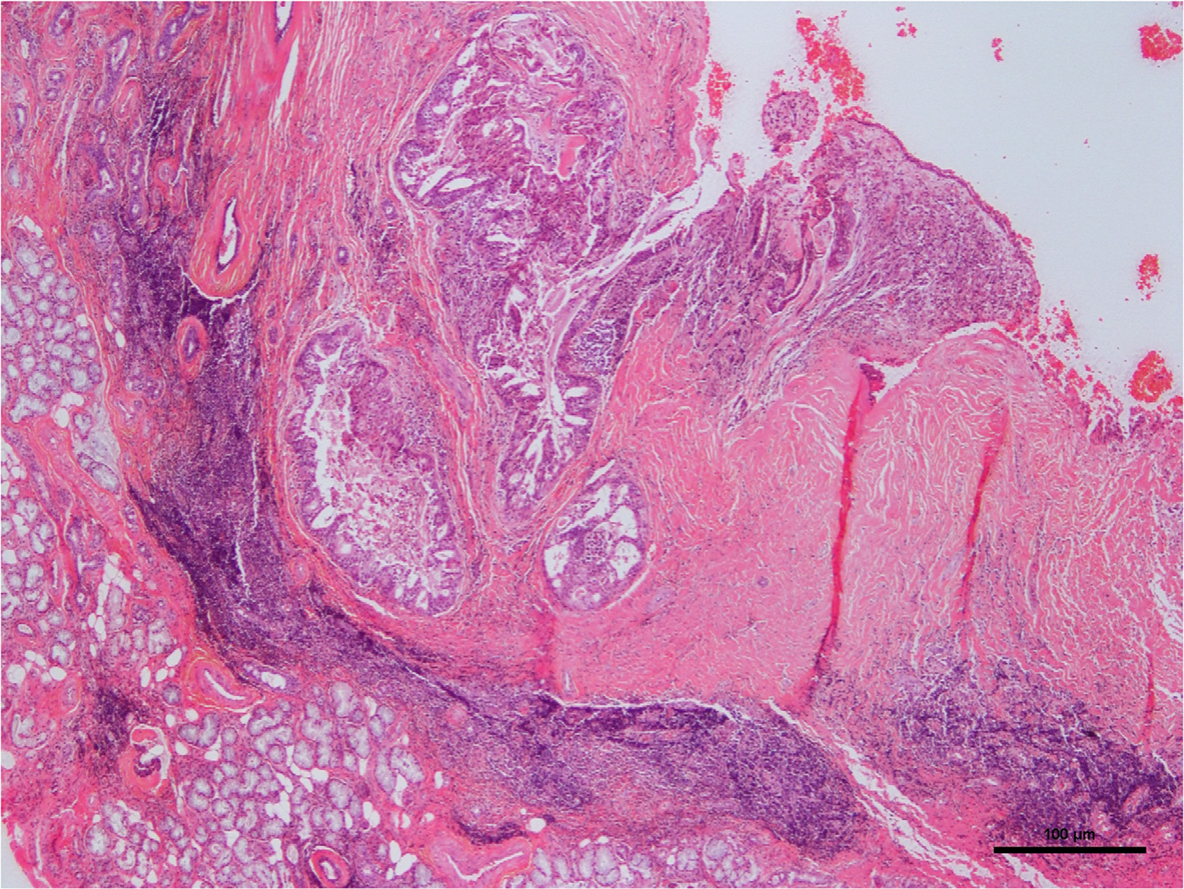
Photomicrograph of hematoxylin-and-eosin-stained section of mucoepidermoid carcinoma.

**Figure 4:**
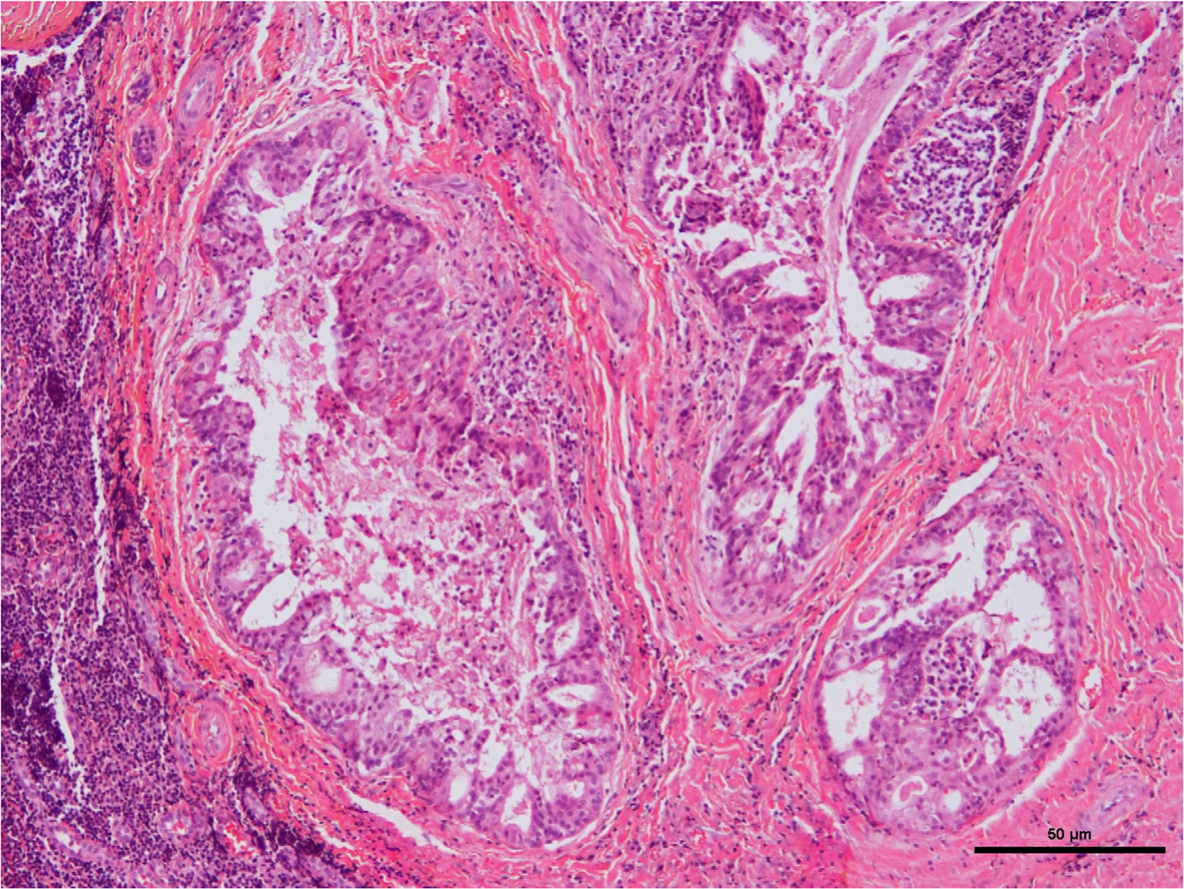
Photomicrograph of hematoxylin-and-eosin-stained section of mucoepidermoid carcinoma.

**Figure 5:**
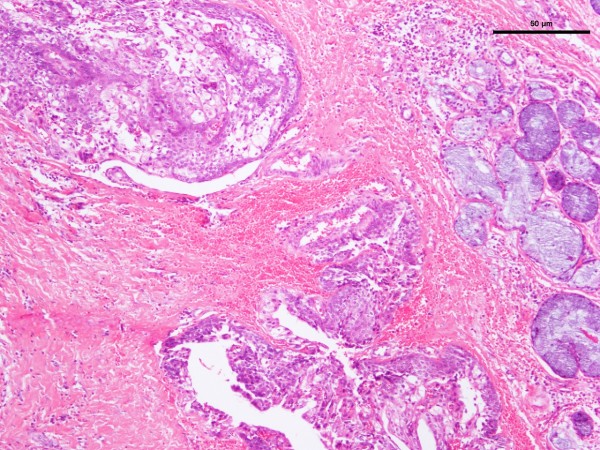
Photomicrograph of hematoxylin-and-eosin-stained section of mucoepidermoid carcinoma.

**Figure 6:**
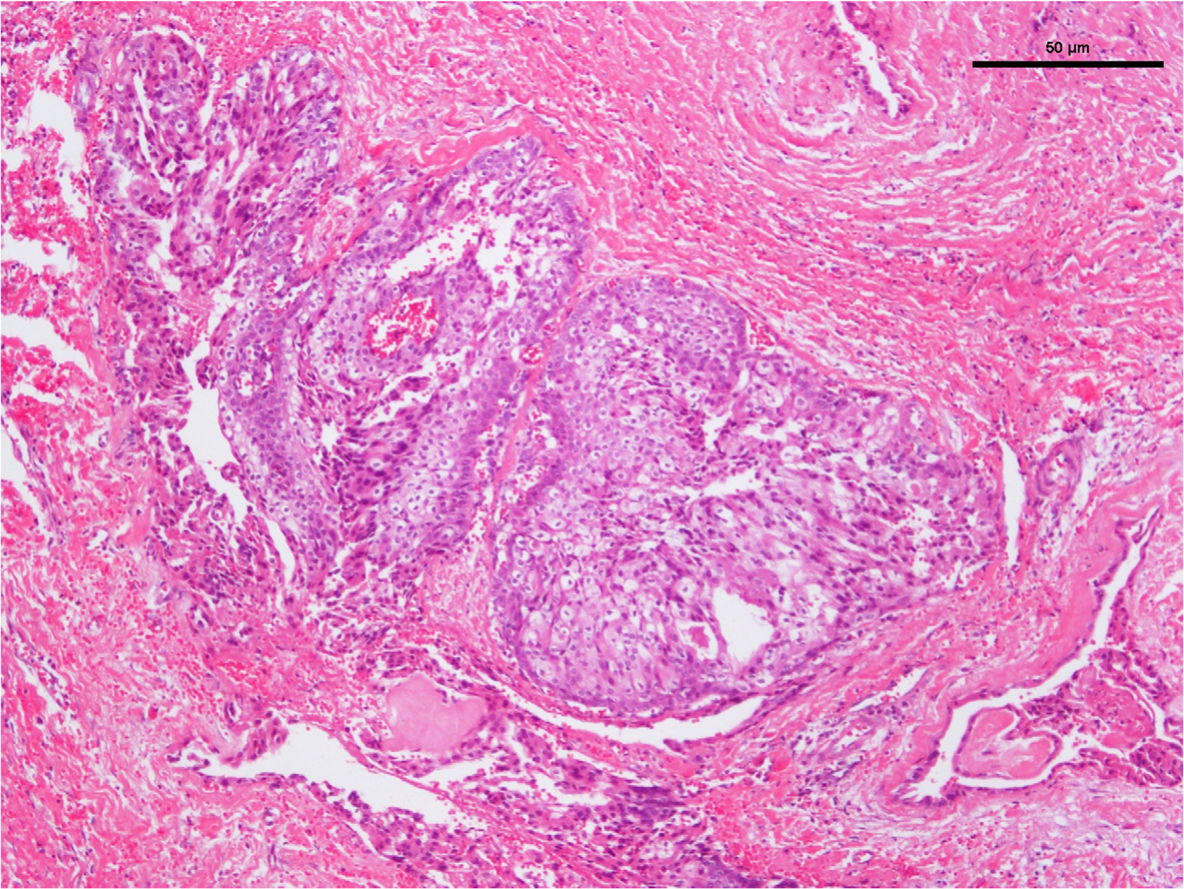
Photomicrograph of hematoxylin-and-eosin-stained section of mucoepidermoid carcinoma.

The age range of the 20 patients was one and a half to 19 years, the mean was 13.5 years, and peak incidence was at the age of 14 years (Figure 1). Five (25%) occurred in patients from birth to 12 years of age but only two of those were in the first decade of life (Figure 1). Of the 11 patients for whom race was known, nine (82%) were white and two (18%) were black. The female-to-male ratio was 2.3:1. The most common locations for the 20 tumors were the hard or soft palate or both (85%), the buccal mucosa (10%), and the upper lip (5%). The site distribution of the lesions is shown in Figure 2. Detailed clinical features were often lacking or scant. Most were described as ‘lumps’ or submucosal nodules, 12 were non-ulcerated, three were ulcerated, three were firm to palpation with pink or flesh-colored surfaces, and seven were fluctuant with surface color alterations ranging from a light blue hue to purplish. The size of the tumor, in 13 cases, ranged from 0.5 to 5cm and the mean size was 2cm. Resorption of bone at the site of the lesion occurred in three (15%) cases, bone was not involved in nine (45%) cases, and bone involvement was not stated in eight (40%) cases. In those patients in whom tumor duration was reported, nine had an average duration of five months. Two patients, a 22-year-old man and a 19-year-old man, reported durations of seven years and ‘forever’, respectively. The histologic grade was provided in 19 cases: 12 (63%) low-grade, four (21%) low- to intermediate-grade, two (11%) intermediate-grade, and one (5%) poorly differentiated. Representative lesions stained with hematoxylin and eosin are shown in Figures 3 and 4 at low magnification and in Figures 5 and 6 at high magnification. Treatment was surgical removal in all cases. Surgical modalities varied from case to case, depending, in part, on the local extent of tumor involvement with the soft tissues or bone (or both), lymph node status, and the tumor’s histologic grade (Table 1).

Thirteen cases had adequate follow-up ranging from three to 21 years, and the mean follow-up duration was 7.9 years. One case from the literature reported recurrence, to indicate a recurrence rate of 7.7% for the 20 cases reviewed. There were no cases of death or metastasis in this series.

## Discussion

Epithelial neoplasms originating in the minor salivary glands account for approximately 15% of all salivary gland neoplasms [6,38]. It has been estimated that about 1% to 5% of all salivary gland tumors develop in children and adolescents [1,8], and MEC is the most common malignancy [5,9,39,40]. In the current LSUSD series, 3.5% of the epithelial minor salivary gland neoplasms occurred in patients 19 years of age or younger; this is in close agreement with the series reported by Waldron *et al*. [38] and Kusama *et al*. [41], who found incidences of 3.7% and 5.4%, respectively. A total of five malignant salivary gland tumors, all MECs, represented 1.3% of all salivary gland tumors accessioned in the LSUSD oral biopsy service from a 35-year period, and this supports the conclusion in the literature that MEC is the most common malignancy of minor salivary glands in the first and second decades of life.

This study combined data from five LSUSD cases and 15 cases from the literature for data analysis. The majority of epithelial salivary gland neoplasms occur late in childhood, after 10 years of age [42]. MECs are generally found between the ages of 10 and 16 years [40,43], which is in general agreement with this case report. In the 20 cases reviewed, 16 MECs occurred in the second decade and the overall average age was 13.5 years. We have included the case reported by Tipton [10] of a 20-month-old with a poorly differentiated MEC; however, in view of its poor documentation, we are not convinced that this tumor is an MEC. Nevertheless, we have included it since it has been frequently referenced as such. According to Mehta and Willging [40], MEC is the most common radiation-induced salivary gland tumor in children. None of the patients in this review had a history of radiation.

Among the 20 cases, there was a female predilection of 2.3:1. Since race was known in only 55% of the 20 cases, no further analysis of this demographic feature was undertaken. This series confirmed that the hard or soft palate (or both) is by far the most common site for intraoral minor salivary gland MECs, followed by the buccal mucosa [39].

The histologic grade of the MEC often reflects the clinical manifestations of the tumor. Intraorally, low-grade MECs tend to be asymptomatic enlargements of prolonged duration. In this study, the average duration was five months before diagnosis; one case had a duration of seven years. Interestingly, seven of the low-grade MECs appeared as fluctuant light blue or purplish submucosal lumps, thus resembling the reactive salivary gland mucocele (mucous retention phenomenon). The reason they possess similar clinical appearances is that low-grade MECs and mucoceles possess mucous cyst formation and mucous pseudocyst formation, respectively. As Flaitz [17] has pointed out, the differential diagnosis for a compressible or fluctuant light blue mass in an intraoral salivary gland-bearing area in a child or adolescent should include reactive and neoplastic lesions, and MEC and mucocele should be at the top of the list. Although MECs are considered rare in the children-adolescent age group, they must be considered when a lesion appears to be similar to a mucocele but is found at a site other than the lower lip mucosa [17].

Histologically, MECs are divided into low-, intermediate-, and high-grade types, which correlate to clinical behavior. Our analysis of the five LSUSD cases was consistent with that of several other studies [1,11-19] in that all of the low- to intermediate-grade MECs originating from intraoral minor salivary glands had a very low recurrence rate and a high survival rate (100%). Recurrence in this series was less than 10%. This is in keeping with the general consensus that low- and intermediate-grade MECs have an indolent clinical course and a minimal chance for metastasis [14]. The current series supports the opinions that MEC in children appears to be somewhat more innocuous than in adults and that the probability of death for children with low-grade MEC is essentially zero [44]. However, others believe that malignant salivary gland malignancies in children exhibit biologic behavior similar to those occurring in adults and therefore require the same treatment principles as those occurring in adults [45,46]. Nevertheless, close clinical follow-up should be long-term, as outlined by April *et al*. [14], because low- to intermediate-grade MECs in this age group can recur many years after initial removal [1,14].

The results of this study and others [17-19] suggest that low- to intermediate-grade MECs originating from intraoral minor salivary glands in children and adolescents can be effectively managed by wide local surgical excision that ensures tumor-free surgical margins. Wide surgical excision in combination with bone removal is preferred only when there is gross periosteal involvement or bone erosion by the MEC [17,18].

It is most likely that the treating dentist would take an intraoral radiograph or an orthopantomograph or both at the time of the initial clinical presentation. However, the treating oral surgeon would need a computed tomography scan to establish the extent of the lesion prior to surgical exploration. Prognosis of the lesion on the basis of imaging modalities has not been investigated, and to date, the only prognostic indicator is the histopathologic grading of the lesion [14].

## Conclusions

MECs have a female predilection and are decidedly uncommon in the first decade of life. MECs have a high predilection for the hard or soft palate or both. Fluctuance and a light blue color are helpful diagnostic clinical clues. MEC must be considered in the differential diagnosis of a lump or mass in a salivary gland-bearing area, especially the palate. The histologic grades of most MECs in the first and second decades of life are low and, to a lesser degree, intermediate. Complete surgical excision is the initial treatment of choice and results in a recurrence rate of less than 10% in this series.

## Abbreviations

LSUHSC, Louisiana State University Health Sciences Center; LSUSD, Louisiana State University School of Dentistry; MEC, mucoepidermoid carcinoma.

## Competing interests

The authors declare that they have no competing interests.

## Authors’ contributions

PR helped to review the clinical and histopatholgic data from the selected cases and the literature and to analyze the data. RBB helped to review the clinical and histopatholgic data from the selected cases and the literature and to analyze the data, reviewed histopathologic microslides, and confirmed the diagnoses for the cases from the LSUSD series. KGC provided the photomicrographs of the histopathologic slides. All authors read and approved the final manuscript.
